# The Double-Edged Influence of Self-Expansion in the Metaverse: A Two-Wave Panel Assessment of Identity Perception, Self-Esteem, and Life Satisfaction

**DOI:** 10.1089/cyber.2022.0400

**Published:** 2024-01-08

**Authors:** Soeun Yang, Haesoo Kim, Minwoo Song, Seunghyun Lee, Jeong-woo Jang

**Affiliations:** ^1^ Institute of Communication Research, Seoul National University, Seoul, Korea.; ^2^ Bowers College of Computing and Information Science, Cornell University, Ithaca, New York, USA.; ^3^ School of Computing, Korea Advanced Institute of Science and Technology (KAIST), Daejeon, Korea.; ^4^ Department of Electrical Engineering and Computer Science, Daegu Gyeongbuk Institute of Science & Technology (DGIST), Daegu, Korea.; ^5^ School of Digital Humanities and Computational Social Sciences, Korea Advanced Institute of Science and Technology (KAIST), Daejeon, Korea.

**Keywords:** Metaverse, social VR, self-expansion, identity disjunction, self-discrepancy, self-esteem, life satisfaction

## Abstract

This study researches the impact of self-expansion experiences in the Metaverse on users' identity perception, self-esteem, and life satisfaction. To do so, the researchers conducted a two-wave panel study with a 3-month interval (*N* = 486) in VRChat, one of the most popular social virtual reality (VR) platforms. As predicted, the increase in self-expansion experience in VR environments positively predicted users' self-esteem and life satisfaction. However, when self-expansion led to a loss of coherency in the self-concept by causing identity disjunction or self-discrepancy, it damaged self-esteem and life satisfaction, respectively. The current findings exhibit that experimenting with and enlarging identity through immersive experiences in the Metaverse could benefit the individual, but only when it does not cause a disconnection between virtual and offline identities. This article discusses the potential opportunities and risks in the Metaverse, emphasizing the importance of advancing our understanding of the self-expansion experience in immersive media.

## Introduction

Following the rise of social virtual reality (VR) technologies, a postreality universe, often referred to as the Metaverse, has risen to prominence. In social VR platforms such as VRChat, Horizon Worlds, and Rec Room, users navigate 3D virtual spaces while engaging in an immersive real-time social experience through the help of VR head-mounted displays,^1,2^ creating and designing new identities through embodied avatars.^3^

Among the core experiences of the Metaverse is self-expansion,^4^ the experience of going beyond and broadening the sense of self.^5^ Individuals are drawn to self-expansion out of a willingness to facilitate self-improvement,^6^ or seeking relief from the stress of self-regulation and social constraints.^7,8^ The Metaverse offers immersive experiences that go beyond the physical world, allowing users to experiment with alternate identity representations^9^ and providing an opportune space for self-expansion.^3,10–12^ Building on the self-expansion model,^5,6,13,14^ this article proposes that self-expansion in the Metaverse will have a beneficial impact on self-esteem and life satisfaction by allowing individuals to overcome the limitations of the physical self.

However, self-expansion in the Metaverse may have a double-edged effect.^15^ As the Metaverse involves deep immersion and involvement with their alternate selves,^16,17^ it could cause identity confusion, negatively impacting self-esteem and life satisfaction. This article proposes that if self-expansion causes the virtual and real identities to become too distant (identity disjunction) or to feel that the virtual self is superior (self-discrepancy), it could lower self-esteem or life satisfaction. To elucidate how self-expansion in the Metaverse predicts mental benefits and how identity dynamics mediate these effects, this study utilized a two-wave panel survey with a 3-month interval in VRChat.

## Literature Review

### Self-expansion in the Metaverse and mental benefits

The self-expansion model states that people constantly try to expand their boundaries of the self to move closer to a state of wholeness.^6^ Individuals seek new relationships, experiences, and activities that grant additional resources to further expand their self.^5,6,13,14^ Previous article recognizes diverse pathways of self-expansion: reading stories^7^; using smartphones to access information, social media, or games^18,19^; and surfing the Internet.^20^ The TEBOTS (temporarily expanding the boundaries of the self) framework describes how temporary media-based self-expansion experiences can lead to mental benefits by alleviating the stress of managing personal and social identities.^7,8^

By providing opportunities for identity experimentation, the Metaverse can further promote self-expansion. Essential motivations for social VR use include expressing one's true self and gratifying self-related needs.^3^ Users can freely define themselves through avatars and find ways to improve or express specific identities,^11,21^ and can also obtain novel experiences, thanks to the increased freedom of activity and spatial presence.^10^ Thus, the Metaverse affords a more immersive path to self-expansion by allowing users to escape the physical constraints of reality.

As self-expansion addresses a fundamental human need for self-improvement, it contributes to overall well-being and mental health.^22–24^ In particular, self-expansion in the Metaverse can facilitate personal growth^18^ and increase self-esteem.^2^ Social VR use has been observed to increase self-esteem in elderly people^25^ or LGBTQ+ individuals^26^ by providing a safe space for social experimentation.

The social nature of the Metaverse also allows the newly constructed virtual selves to be supported by peers who accept their identity.^11,21^ Similarly, people engaging in self-expanding activities in social VR may experience an increase in confidence and self-worth.^2^ This, in turn, can lead to an increase in self-esteem, as individuals feel more capable, competent, and in control of their lives.

Considering that self-esteem plays a key role in shaping individuals' perceptions of their lives,^27,28^ increase in self-esteem through self-expansion can also heighten overall life satisfaction. Previous research has found self-esteem to predict life satisfaction across various contexts.^29,30^ For instance, experiences such as seeking information on the Internet^31^ or receiving positive reactions on social media^32^ can enhance life satisfaction through the mediating role of self-esteem. This article argues that self-expansion in the Metaverse can directly contribute to improvement in self-esteem, which leads to an increase in life satisfaction.

**H1: Self-expansion in the Metaverse positively predicts self-esteem.**

**H2: The increase in self-esteem in the Metaverse positively predicts life satisfaction.**

### The influence of self-expansion on identity dynamics and self-concept

Alternatively, self-expansion in the Metaverse might yield adverse effects to one's self-concept and identity. Although findings are mixed, previous studies have examined a possible trade-off between self-expansion and one's clarity of self,^15,33^ such as in the self-concept fragmentation hypothesis.^16,17^ Thus, adding more facets to one's self may cause the self-concept to get too complex, creating a disconnect between the identities. In this article, this will be referred to as *identity disjunction*, where various representations of one's identity are perceived as separate entities with no overlap. This study argues that self-expansion in the Metaverse can exacerbate identity disjunction due to the ease of crafting new identities and the lack of dependence on existing identities.

What may also occur is a perceived comparison between the offline and virtual personalities. When individuals consider their virtual selves to be superior to their actual selves, it promotes feelings of *self-discrepancy*. Suh examined self-discrepancy in VR users by comparing their self-appraisal of their virtual and online selves on various attributes (e.g., intelligence, expertise, and sociability), observing that VR users with higher self-discrepancy were more committed to their virtual characters and community.^34^

Jin also found that virtual self-discrepancy occurs when individuals continuously alternate between virtual and offline identities.^35^ Individuals often seek to improve themselves by customizing their virtual representations to be more conventionally attractive (i.e., wishful identification).^36,37^ Active self-expansion could heighten such disparity in the self-appraisal of the two selves, leading to feelings of self-discrepancy.

**H3a-b: Self-expansion in the Metaverse may predict (a) identity disjunction and (b) feelings of self-discrepancy.**

### Impact of virtual identity dynamics in self-esteem and life satisfaction

This article predicts that self-expansion generally provides psychological benefits (H1 and H2), whereas it may also cause psychological ill effects through identity disjunction (H3a) and virtual self-discrepancy (H3b), as it may challenge the coherence and integrity of the overall self-concept.^38–40^ Previous research have consistently identified positive relationships between identity clarity and self-esteem, proposing that a clearly defined, stable, and internally consistent self-concept contributes to psychological adjustment.^38^

This implies that identity disjunction caused by excessive self-expansion may result in poor psychological adjustment.^4,17^ Similarly, identity discrepancy theory posits that discrepancies between various social roles cause psychological distress.^41^ This may also occur in virtual identities, as people often perceive their virtual selves to be superior to their actual selves,^42,43^ which can cause doubts about one's self and lower their self-esteem.^16,17^

Thus, this article argues that both identity disjunction and self-discrepancy will negatively predict users' self-esteem. This would ultimately lead to a serial mediation effect, where self-expansion experience will exert a negative indirect effect on self-esteem, and on life satisfaction through self-esteem.

H4a-b: (a) Identity disjunction and (b) self-discrepancy in the Metaverse negatively predict self-esteem.

## Methods

### Sample

**FIG. 1.**
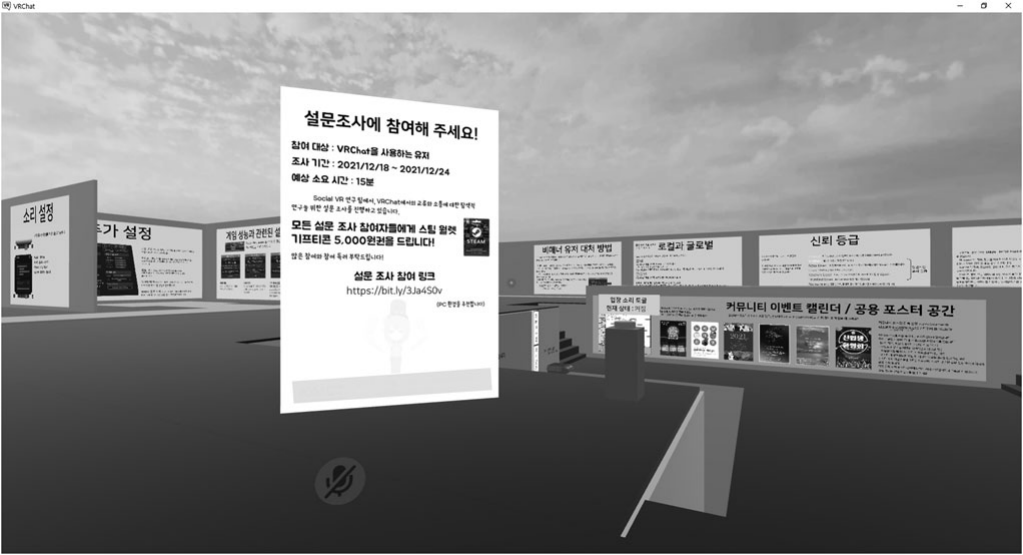
Poster displayed in the VRChat.

An online survey was conducted as a two-wave panel study with a 3-month interval (W1: December 2021, W2: March 2022). Participants >19 years were recruited by displaying a poster in a Korean VRChat Tutorial Community (Fig. 1). VRChat was selected as the target platform given its prominence in the social VR community, recording >20k average Steam users per month in 2022. This was significantly larger than any other social VR service, for example, Rec Room (∼2k users/month) or Neos VR (∼200 users/month).

Participants received a $10 Steam gift card in exchange for voluntary participation. The survey recorded 789 valid responses at wave 1 and 494 at wave 2 (retention rate: 62.6 percent). Excluding cases where the identification codes in the 2 waves did not match, 486 users participated in both waves (see Table 1 for demographics). This study was approved by the IRB of Korea Advanced Institute of Science and Technology.

**Table 1.** Sample Characteristics

	M (*SD*) or percentag
Gender, male	77.16
Age^a^	
1: 10s	28.36
2: 20s	65.15
3: 30s	6.49
Education	
1: Middle school graduates	19.91
2: High school graduates	26.30
3: Attending or graduated a vocational/professional program	18.48
4: Attending or graduated an undergraduate program	33.65
5: Attending or graduated a graduate program	1.66
Income (annual household income)	
1: <10,000,000 KRW	7.46
2: 10,000,000–30,000,000 KRW	19.66
3: 30,000,000–50,000,000 KRW	28.81
4: 50,000,000–70,000,000 KRW	18.98
5: 70,000,000–90,000,000 KRW	10.51
6: ≥90,000,000 KRW	14.58
Time spent on VRChat, hours^b^	
Wave 1	1,863.97 (2,399.04)
Wave 2	2,425.61 (3,722.217)

^a^ Participants selected from the provided age ranges in intervals of 10.

^b^ The total time usage recorded and displayed in VRChat.

*SD*, standard deviation.

### Measures

All measures were anchored on a 7-point Likert scale (1 = *Strongly disagree*, 7 = *Strongly agree*) (see Table 2 for summary statistics). To measure *self-expansion* experience, four items were adopted from previous research on social VR^10^ (see Appendix A1 for measurement items). For *identity disjunction*, the pictorial inclusion of the other in the self scale was used.^44^ Seven pictures presenting varying degrees of overlap between two circles were provided, asking the respondents to indicate which best represents the relationship between their offline and virtual selves.

**Table 2.** Descriptive Statistics of the Key Variables

Variables	M	*SD*_overall_	*SD*_wihtin_	*SD*_between_	Min	Max	Skewness	Kurtosis	α	ICC
Self-expansion	4.71	1.35	0.61	1.20	1	7	−0.34	2.88	0.76	0.59
Identity disjunction	3.69	1.95	0.95	1.70	1	7	0.23	1.91	–	0.53
Self-discrepancy	0.12	1.26	0.63	1.09	−4.6	6	−0.05	5.29	0.72	0.50
Self-esteem	4.54	1.61	0.61	1.49	1	7	−0.37	2.44	0.84	0.71
Life satisfaction	4.28	1.43	0.59	1.31	1	7	−0.14	2.56	0.98	0.66

ICC, intraclass correlations.

This item was reverse-coded, higher scores representing greater identity separation. For *self-discrepancy*, the survey adopted Suh's scale, subtracting the self-appraisal value of the offline self from that of the virtual self, potential values ranging from −6 to 6.^34^ A higher self-discrepancy score indicates that participants perceived the characteristics of their virtual self as superior to those of the real self. *Self-esteem* was measured by Rosenberg's scale.^45^ Finally, *life satisfaction* was measured with three items from Diener's scale.^46^

### Analysis

To test the hypotheses, this article utilized a multilevel structural equation modeling (MSEM) using Mplus 7.4. MSEM differs from multilevel modeling (MLM) by directly partitioning the between- and within-person variances of each variable. By analyzing within-person changes under controlled conditions of unobserved heterogeneity, MSEM identifies nonspurious relationships,^47^ while simultaneously estimating the suggested direct and indirect effects at the within- and between-person levels.

For MSEM, the data were organized in long format, with the within-person (level 1) and the between-person level (level 2), consisting of 972 cases at level 1 and 486 cases at level 2. Multiple-item variables were aggregated by computing their average scores and included as observed variables. Using the observed variables, MSEM established latent variables at the between- and within-person level to examine the relationships among factors and their mixed properties at each respective level.

Covariates such as age, gender, education, and income, which were measured at wave 1, were treated as between-subject factors. Conversely, time spent in VR was considered as both between- and within-subject factors, similar to other time-varying variables. To account for skewed distribution, time spent in VR was transformed using its square root.

To determine the appropriateness of employing an MLM approach, intraclass correlations (ICCs) were calculated for each variable.^47–49^ As presented in Table 2, all ICC values exceeded 0.5, indicating substantial between- and within-person variation. The MLR estimator was employed to obtain more accurate parameter estimates less affected by non-normality^50^ and utilized full information maximum likelihood estimation to handle missing data.

Before examining the MSEM, we conducted a confirmatory factor analysis to evaluate the measurement model that included latent factors of self-expansion, self-discrepancy, self-esteem, and life satisfaction with their corresponding indicators. Identity disjunction was excluded from this model because it was operationalized with one observed variable. The measurement model had an acceptable fit, *χ*^2^ (71) = 535.40, *p* < 0.001, the goodness-of-fit index = 0.93, the comparative fit index (CFI) = 0.92, the Tucker-Lewis index (TLI) = 0.90, the root mean square error of approximation = 0.08, with standardized factor loadings >0.54, and all latent variables had composite reliability values >0.7.

## Results

**FIG. 2.**
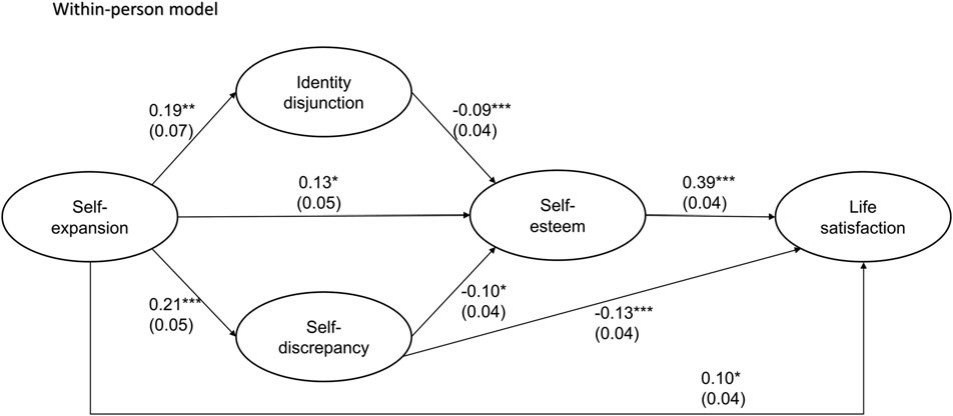
Path coefficients of the estimated within-person model: The relationships among self-expansion, identity perception, self-esteem, and life satisfaction. Values in *parentheses* are standard errors. *p < 0.05, **p < 0.01, and ***p < 0.001.

The MSEM of this study yielded adequate to excellent fit, *χ*^2^ (60) = 737.89, *p* < 0.001, CFI = 1.00, TLI = 1.00, SRMR_within_ = 0.00, SRMR_between_ = 0.00. We tested the hypotheses through a within-person model to see the relations among time-varying factors (Table 3). In the estimated within-person model, self-expansion in the Metaverse positively predicted life satisfaction through self-esteem, supporting H1 and H2 (see Fig. 2 for direct paths; Table 4 for indirect paths). In addition to the indirect effect of self-expansion on life satisfaction through self-esteem, the results revealed that self-expansion directly predicted life satisfaction.

**Table 3.** Unstandardized Estimates and Standard Errors for the Within-Person Model of Multilevel Structural Equation Modeling

	Identity disjunction			Self-discrepancy			Self-esteem			Life satisfaction		
	Estimate	SE	*p*	Estimate	SE	*p*	Estimate	SE	*p*	Estimate	SE	*p*
Time spent on VRChat	0.00	0.01	0.56	0.00	0.00	0.48	0.01^***^	0.00	<0.001	0.00	0.04	0.71
Self-expansion	0.19^**^	0.07	0.01	0.21^***^	0.05	<0.001	0.13^*^	0.05	0.02	0.10^*^	0.04	0.02
Identity disjunction							−0.09^***^	0.04	<0.001	−0.01	0.03	0.73
Self-discrepancy							−0.10^*^	0.04	0.02	−0.13^***^	0.04	<0.001
Self-esteem										0.39^***^	0.04	<0.001

^*^ *p* < 0.05; ^**^p < 0.01; ^***^p < 0.001.

SE, standard error.

**Table 4.** Estimates of Within-Person Indirect Effects

Path	Indirect effect	95% confidence interval	
		Lower bound	Upper bound
Self-expansion→Self-esteem→Life satisfaction	0.05	0.01	0.09
Self-expansion→Self-discrepancy→Life satisfaction	−0.03	−0.05	−0.01
Self-expansion→Identity disjunction→Self-esteem→Life satisfaction	−0.01	−0.01	−0.0002
Self-expansion→Self-discrepancy→Self-esteem→Life satisfaction	−0.01	−0.02	−0.0003

Meanwhile, self-expansion showed a negative association with identity disjunction and self-discrepancy, supporting H3a and H3b. This led to lower self-esteem, in line with H4a and H4b. The negative effects of self-expansion on self-esteem, mediated by virtual-physical self-separation (i.e., identity disjunction and self-discrepancy), ultimately influenced lower overall life satisfaction (Table 4). Furthermore, self-discrepancy directly damaged life satisfaction, indicating an indirect effect where self-expansion lowered life satisfaction through increased self-discrepancy. Lastly, the time spent on VRChat, a time-varying covariate, positively predicted self-esteem, but did not predict identity disjunction, self-discrepancy, or life satisfaction.

**FIG. 3.**
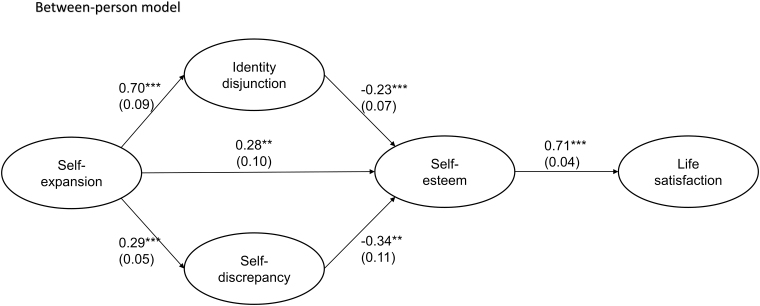
Path coefficients of the estimated between-person model: The relationships among self-expansion, identity perception, self-esteem, and life satisfaction. Values in *parentheses* are standard errors of those path coefficients. **p < 0.01 and ***p < 0.001.

In the between-person model (Fig. 3), which aggregated scores across two-time points to test the associations influenced by individual differences, participants who experienced greater self-expansion showed higher identity disjunction, self-discrepancy, and self-esteem, but not life satisfaction. This demonstrates that, as individuals' self-expansion increases across time, their life satisfaction also increases (within-person model), but those who experience self-expansion do not necessarily report better life satisfaction (between-person model).

Identity disjunction and self-discrepancy both showed a negative association with self-esteem, and self-esteem exhibited a positive association with life satisfaction. Among covariates, age exhibited a positive relationship with identity disjunction, whereas educational attainment showed a negative association. Income was negatively related to self-discrepancy. In addition, gender was a significant predictor of self-esteem, such that male (vs. female) participants exhibited higher self-esteem (Appendix Table A1).

## Discussion

This study researched how self-expansion in the Metaverse affects identity perception, self-esteem, and life satisfaction. As predicted, self-expansion in the Metaverse led to self-esteem and positive changes in self-esteem extended beyond the virtual realm, contributing to overall life satisfaction. However, when self-expansion in the Metaverse triggered a loss of coherency in the self-concept, it reduced self-esteem and life satisfaction. This holds significant implications on the complexity of identity exploration in the Metaverse, and provides insights into how self-expansion could be leveraged so that it does not undermine its mental benefits.

Notably, self-expansion damaged self-esteem when it caused identity disjunction. This indicates that excessive self-expansion could cause fragmentation between identities, deterring the development of a coherent self-concept.^16,17^ Previous research on multicultural identities suggests that it is not the presence of multiple identities but rather the relationship among them that affects mental well-being.^51,52^ People with multiple identities need to integrate and manage various aspects of those identities for better adjustment. This idea that a lack of identity coherence damages self-esteem can similarly be applied to the Metaverse.

Likewise, once self-expansion in the Metaverse triggered self-discrepancy, it led to a decrease in both self-esteem and life satisfaction. This path could be accounted to the self-discrepancy theory, which states that individuals experience psychological distress as their actual selves deviate from their ideal selves.^41,53^ Virtual identities are independent from the preconditions of the real self, and users often utilize this to create improved, more “ideal” versions of themselves.^36,42,43^ As the differences become more prominent, the inadequacies of their offline selves may become more pronounced, resulting in a decrease in life satisfaction.

Our findings also have theoretical significance in reaffirming the existing self-expansion model while proposing new directions for exploration of self-expansion in emerging media, specifically social VR. Previously examined pathways of self-expansion have been mostly focused on external factors, searching for resources outside of the individual that augment the “existing” self^2,5,6,8^ such as relationships,^6^ brand consumption,^13^ story exposure,^7^ and engaging in various activities.^18,19^

However, in the Metaverse, self-expansion experiences also stem from a self-built, alternative representation of their own identity.^10^ This can potentially lead to distinct outcomes of self-expansion specific to immersive virtual spaces, such as a conflict between the newly developed and existing aspects of the self. The results imply that self-expansion in the Metaverse can be beneficial, in accordance with the existing self-expansion model, provided that it preserves one's true identity and does not exacerbate self-discrepancy or identity disjunction.

To apply these findings on a practical level, Metaverse platforms could aim to further facilitate self-expansion, introducing elements such as spatial presence,^10,54^ interactivity,^55^ or various interactions not accessible in real life,^7,8^ to maximize its benefits to users. Platforms should also focus on prolonged maintenance of the expanded self, as reduced usage or inability to access the technology could cause self-contraction and a loss of the expanded self.^56,57^

Moreover, to prevent the negative potential effects of self-expansion to identity, users and platforms should work toward facilitating transfer between the offline and virtual identities. Integrating pre-existing social relationships such as family, work, and school to a virtual modality^58–60^ could work to reduce the perceived divide between these communities. Self-reflection interventions that encourage positive identification between their avatars and their real-life selves could also facilitate the connection between virtual and physical experiences.^61^

### Directions for future research and limitations

The authors encourage future research to explore the generalizability of the current results. Indicators such as psychological well-being^62^ can be examined for a more detailed understanding of the Metaverse's role in different aspects of well-being such as personal growth and self-acceptance. Future article can develop advanced measurement scales to better assess experiences within emerging social VR environments, encompassing multiple dimensions, including personality, appearance, and experience. Finally, future research should examine if the current findings can be generalized to other social VR platforms, and if differences in self-expansion arise according to the unique affordances of each platform.

This study has several limitations. This study used a relatively short interval (3 months), which prevents assessment of longer-term effects. Future research with longer intervals could explore the cumulative or sustained effects of self-expansion in the Metaverse. Moreover, as it was not possible to test temporal order in within-person analysis with two-wave data, this study only examined if one variable systematically changes when another changes over time.

More sophisticated multiwave longitudinal studies (e.g., dynamic panel data model with fixed effects^63,64^) may elucidate these within-person causal effects. The uneven gender distribution of participants may also limit generalizability, and future studies should strive for a more balanced representation. Finally, in the power analysis using Monte Carlo simulations,^65,66^ some paths were found to have insufficient power (<0.8). The lack of significance of the suggested paths could be influenced by the sample size. Based on the current analysis, a larger sample size of 700 would be sufficient to yield more reliable outcomes.

## Conclusion

This study underscores the significance of the relationship between offline and virtual selves in determining the influence of self-expansion. This research agenda should be further explored,^19,20^ encompassing diverse contemporary media that enable the construction of multiple personas.^67–69^ Although self-expansion in the Metaverse provides benefits to self-esteem and life satisfaction, our lives are still primarily offline, exemplifying the importance of the integration and coherence of the expanded selves. Future studies should examine the affordances that facilitate self-expansion and explore how to derive greater benefits while maintaining congruence in one's identity.

## Appendix

### Self-Expansion

1. In VRChat, I experience facing situations and challenges other than those in my own life.
2. In VRChat, I experienced things that I haven't before.
3. In VRChat, I experience what it is like to be someone else.
4. In VRChat, I relate to others in ways different than I normally do myself.

### Identity Disjunction

*Note:* This scale was reverse-coded for identity disjunction.

### Self-Discrepancy

What do you think are you like in the real world?

1. I make friends easily.
2. I tend to be proactive and actively participate in various activities.
3. I help and guide others.
4. I am an expert with special skills and knowledge.

What do you think are you like in VRChat?

1. I make friends easily.
2. I tend to be proactive and actively participate in various activities.
3. I help and guide others.
4. I am an expert with special skills and knowledge.

### Self-Esteem

1. On the whole, I am satisfied with myself.
2. I take a positive attitude towards myself.
3. I feel that I am a person of worth.

### Life Satisfaction

1. I am satisfied with my life.
2. If I could live my life over, I would change almost nothing.
3. The conditions of my life are excellent.

**Appendix Table A1.** Unstandardized Estimates and Standard Errors for the Between-Person Model of Multilevel Structural Equation Modeling

	Identity disjunction			Self-discrepancy			Self-esteem			Life satisfaction		
	Estimate	SE	*p*	Estimate	SE	*p*	Estimate	SE	*p*	Estimate	SE	*p*
Age	0.26^**^	0.10	0.01	0.09	0.06	0.15	−0.14	0.09	0.12	−0.07	0.05	0.21
Gender (0 = male, 1 = female)	0.10	0.09	0.25	−0.01	0.06	0.88	−0.18^*^	0.08	0.02	0.06	0.05	0.20
Income	0.04	0.06	0.53	−0.08^*^	0.04	0.05	0.12^*^	0.06	0.04	0.06	0.04	0.08
Education	−0.22^**^	0.08	0.01	−0.05	0.05	0.33	0.06	0.07	0.43	−0.03	0.04	0.53
Time spent on VRChat	0.00	0.00	0.28	0.01^***^	0.00	<0.001	0.00	0.00	0.63	0.00	0.00	0.28
Self-expansion	0.70^***^	0.09	<.001	0.29^***^	0.05	<0.001	0.28^**^	0.10	0.01	−0.07	0.07	0.30
Identity disjunction							−0.23^***^	0.07	<0.001	−0.01	0.05	0.78
Self-discrepancy							−0.34^**^	0.11	0.003	0.10	0.08	0.19
Self-esteem										0.71^***^	0.04	<0.001

^*^ *p* < 0.05; ^**^*p* < 0.01, ^***^*p* < 0.001.

SE, standard error.
